# Gastroenteropancreatic neuroendocrine neoplasms: epidemiology, genetics, and treatment

**DOI:** 10.3389/fendo.2024.1424839

**Published:** 2024-09-30

**Authors:** Baizhou Tan, Beiyu Zhang, Hongping Chen

**Affiliations:** ^1^ Department of Histology and Embryology, School of Basic Medical Sciences, Jiangxi Medical College, Nanchang University, Nanchang, China; ^2^ Queen Mary School, Jiangxi Medical College, Nanchang University, Nanchang, China; ^3^ Jiangxi Key Laboratory of Experimental Animals, Nanchang University, Nanchang, China

**Keywords:** gastroenteropancreatic neuroendocrine neoplasm, epidemiology, treatment, neuroendocrine tumor, genetics

## Abstract

The incidence of gastroenteropancreatic neuroendocrine neoplasms (GEP NEN) is increasing at a rapid pace and is becoming an increasingly important consideration in clinical care. Epidemiological data from multiple countries indicate that the incidence of gastroenteropancreatic neuroendocrine neoplasms (GEP NEN) exhibits regional, site-specific, and gender-based variations. While the genetics and pathogenesis of some GEP NEN, particularly pancreatic NENs, have been investigated, there are still many mechanisms that require further investigation. The management of GEP NEN is diverse, but surgery remains the primary option for most cases. Peptide receptor radionuclide therapy (PRRT) is an effective treatment, and several clinical trials are exploring the potential of immunotherapy and targeted therapy, as well as combination therapy.

## 1 Introduction

Neuroendocrine neoplasm (NEN) is a heterogeneous group of tumors originating from the diffuse neuroendocrine system (1). NEN can be present in any part of the body and can lead to various hormonal syndromes (2). The gastroenteropancreatic (GEP) NEN represents the most prevalent site of NEN, accounting for approximately 55% to 70% of the total number of NEN cases (3, 4). GEP NEN is primarily observed in the foregut, midgut, and hindgut (5). NEN can manifest in other locations, including the lungs, thymus, parathyroid, thyroid, adrenal glands, and pituitary glands (6). Furthermore, approximately 10% of NEN cases are associated with genetic syndromes, including multiple endocrine neoplasia type 1, von Hippel-Lindau and neurofibromatosis type 1 (7).

The occurrence of GEP NEN is rare (1). However, the global incidence of NENs has increased markedly over the past few decades, particularly in North America, and the clinical significance of NENs is becoming increasingly important (8, 9). The data from Surveillance, Epidemiology, and End Results (SEER) program show a 6.4-fold increase in the incidence of NEN in the United States between 1973 and 2012, and the trend is more pronounced for NEN than for other tumor types (4).In addition, the incidence of high grade GEP NEN increased approximately 5.3-fold between 1988 and 2010 (10). At the time of initial diagnosis, more than 50% of patients with NENs have already developed lymph node metastases (11). The liver was the primary site of metastasis, accounting for 82% of all cases, while the small intestine was identified as the primary source of NEN metastasis (12).

Malignant GEP neuroendocrine tumors were initially described by Oberndorfer as carcinoid due to their distinctive clinical characteristics, which have been observed for over a century (8). Nevertheless, the term carcinoid is still employed, occasionally resulting in some ambiguity.

NEN can be classified as either functional or non-functional, depending on whether it produces hormones. The percentage of functioning pancreatic NET (PNET) is estimated to be between 30 and 40% (13). Most tumors are non-functional, whereas functional NEN can produce hormones such as insulin, gastrin, serotonin, glucagon, etc., which can lead to different clinical symptoms (14–16). Tumor burden and the primary site of the tumor can also affect clinical signs and symptoms (17).

Over the past 10 years, methods and techniques for the classification, diagnosis and treatment of NENs have advanced significantly (4, 18). Currently, the main treatments for GEN NEN are surgery, peptide receptor radionuclide therapy, chemotherapy, and newer treatments such as immunotherapy and targeted therapy are still being developed, and combination therapies have become popular in the treatment of GEP NEN. This review focuses on the epidemiology, genetics, and treatment of GEP NEN.

## 2 2022 WHO classification

There has been confusion over the naming and classification of NEN (19). The World Health Organization (WHO) has established the most frequently used system for classifying NEN, which provides a new nomenclature for NEN (20). The most recent updates are the 2019 and 2022 versions.

The WHO classifies neuroendocrine neoplasms NEN into two categories: neuroendocrine tumors (NET) and neuroendocrine carcinomas (NEC). While NET is characterized by well-differentiated neuroendocrine cells, NECs exhibit less pronounced differentiation. These two types not only exhibit disparate pathological, morphological, and molecular characteristics but also manifest distinct epidemiological, clinical, therapeutic, and prognostic features.

In 2022, WHO published the latest version of the classification of GEP NEN (**Table 1**). Based on whether the tumor secretes hormones and causes characteristic clinical manifestations it is classified as non-functional and functional.

**Table 1:** The 2022 WHO epithelial neuroendocrine neoplasms classification for Gastrointestinal tract and pancreato-biliary tract (21).

Neuroendocrine Neoplasm	Classification	Mitotic Count and Ki-67 Index	Other characteristics
Well-differentiated neuroendocrine tumor (NET)	NET,Grade 1	<2 mitoses/2 mm^2^ and/or Ki-67 <3%	
	NET,Grade 2	2-20 mitoses/2 mm^2^ and/or Ki-67 3 - 20%	
	NET,Grade 3	>20 mitoses/2 mm^2^ and/or Ki-67 > 20%	
Poorly differentiated neuroendocrine carcinoma (NEC)	Small cell NECs	>20 mitoses/2 mm^2^ and/or Ki-67 >20% (often > 70%)	Small cell cytomorphology
	Large cell NECs	>20 mitoses/2 mm^2^ and/or Ki-67 > 20% (often >70%)	Large cell cytomorphology
Well- or poorly-differentiated mixed neuroendocrine-non-neuroendocrine neoplasm (MiNEN)	Variable	Both are variable	

GEP, Gastroenteropancreatic; NEC, neuroendocrine carcinoma; NET, neuroendocrine tumor.

NET is typically classified as low-grade (G1), intermediate-grade (G2), or high-grade (G3) based on their proliferation rate, as indicated by the Ki-67 index and mitotic count. NET G1 is characterized by a low proliferation rate, with a Ki-67 index typically below 3%, and is classified as the least aggressive among neuroendocrine neoplasms.NET G2 demonstrates a moderate proliferation rate, with a Ki-67 index ranging from 3% to 20%, and is more aggressive than G1 but less so than G3. NET G3 is highly aggressive, with a Ki-67 index exceeding 20%, reflecting a high rate of cell division and the most aggressive behavior within the NET.

In contrast, NECs are characterized by a high proliferative rate and a rapid growth pattern, and are classified as tumors. NEC can be categorized into small cell type and large cell based on the morphology of tumor cells. The WHO Classification of Endocrine Tumors, 5th edition, has adopted these classification principles (21).

However, some studies are still using outdated WHO classifications such as the 2010 version. Crucially, what is now graded as grade 3 NET are still considered NECs, since grade 3 was specifically designated as NECs in the 2010 WHO classification (22).

## 3 Epidemiology

**Figure 1:**
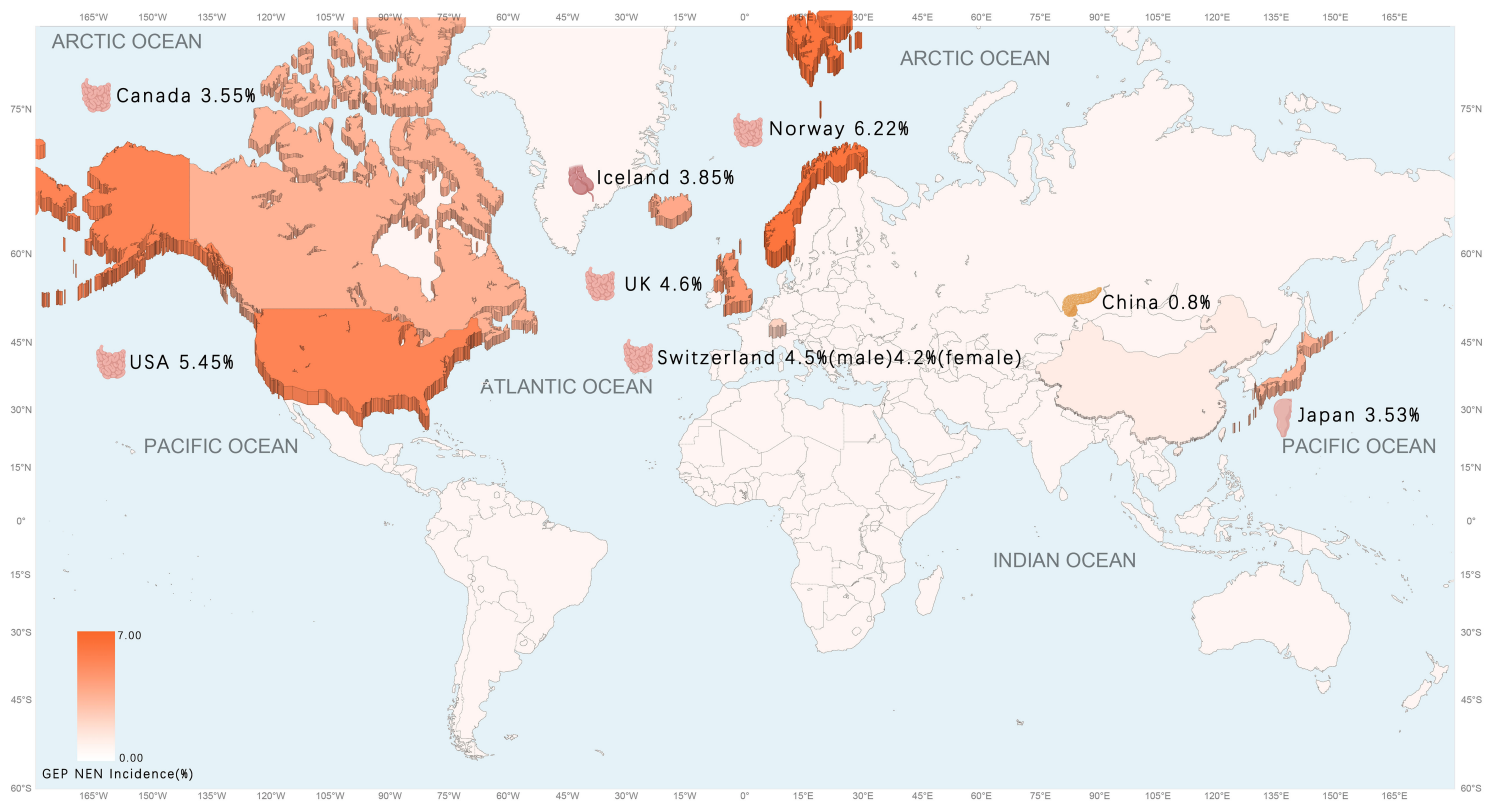
The age-adjusted incidence of GEP NEN in the world map.

As evidenced by pertinent research, the prevalence of GEP NET is on the rise globally, particularly in North America (23). The incidence of GEP NET exhibits considerable variation across countries and regions, with notable differences in the most common sites of cancer (**Table 2**) (**Figure 1**). Small intestinal NET (SiNET) and rectum NET are most common in North America (9). In Asia, the incidence of rectal and pancreatic NET is highest, and in Europe, the most common NET are small intestine and appendix (27–31) (**Table 3**). Overall survival rates for patients with GEP NET seem to be improving in recent years (23). The increased prevalence of GEP NEN is attributed to recent improvements in diagnostic techniques and histological classification, particularly in the rectum, stomach, and pancreas (31, 32). NET can occur at all ages, except for appendiceal tumors, which occur mainly after 50 years of age, and hereditary syndromes can develop earlier (16).

**Table 2:** The age-adjusted incidence of GEP NEN, by country.

Country	GEP NEN Incidence (Cases per 100,000)	Period	Reference
USA	5.45	2015	(9)
Canada	3.55	2009	(24)
Switzerland	4.5(male)4.2(female)	2011–2016	(25)
UK	4.6	2013–2015	(26)
Norway	6.22	2017–2021	(27)
Iceland	3.85	2000–2014	(28)
China	0.8	2017	(29)
Japan	3.53	2016	(30)

**Table 3:** Common primary sites of GEP NET, by country.

Country	First	Second	Third	Reference
USA	Small intestine	Pancreas	Stomach	(9)
Canada	Small intestine	Large intestine (including appendix)	Rectum	(24)
Switzerland	Small intestine	Appendix	Pancreas	(25)
UK	Small intestine	Pancreas	Appendix	(26)
Norway	Small intestine	Appendix	Pancreas	(27)
Iceland	Appendix	Jejunum/Ileum	Stomach	(28)
China	Pancreas	Stomach	Rectum	(29)
Japan	Rectum	Pancreas	Stomach	(30)

A gender-based disparity in GEP NET survival has been observed, with females exhibiting a higher survival rate than males, irrespective of staging and morphology. In most digestive system organs, females demonstrated enhanced survival rates, which reached statistical significance (33). Male pancreatic NET is larger than female pancreatic NET, and the rate of undifferentiated and poorly differentiated is higher, but there is no significant difference in distant metastasis rate. Overall survival differences are found in the early stage, but not in stage 3 or 4 disease (34). Another study demonstrated that women with GEP NET exhibited a higher prevalence of G1 stage tumors, yet comparable proliferation rates to men. There were no notable discrepancies in the metastatic rate or metastatic site, and no discernible differences in treatment by gender (35).

### 3.1 The United States

In a cohort study comprising over 40,000 patients with GEP NET, most patients had NEN in the rectum and small intestine. The age-adjusted incidence rate of GEP NET exhibited a notable upward trajectory from 1975 to 2015, with a ratio of 1.05 per 100,000 in 1975 and 5.45 per 100,000 in 2015. The most common primary tumor sites for GEP NET were the rectum (28.6%), the small intestine (28.1%), and the pancreas (16.4%) (9). A paper based on the SEER database indicates that the incidence of pancreatic neuroendocrine tumors (PNET) in the USA has increased with age from 1975 to 2018 (36). The highest rates of GEP NET are found in African Americans, with men having the highest incidence of high-grade tumors (37).

### 3.2 Canada

The incidence of GEP NET in Canada increased from 1.18 per 100,000 in 1994 to 3.55 per 100,000 in 2009. The most common sites of primary NET overall were the lungs (25.0%), the small intestine (18.1%), the large intestine (12.9%), and the rectum (12.3%) (24).

### 3.3 Switzerland

The incidence of GEP NET increased consistently between 1976 and 2016, with no significant gender differences, but the exact reason for the rise is unknown (25). The incidence of GEP NEN has increased for both genders. For males, the rate rose from 2.4 per 100,000 individuals between the periods of 1976-1980 to 4.5 per 100,000 individuals between 2011-2016. For females, the rate increased from 2.3 per 100,000 individuals between the periods of 1976-1980 to 4.2 per 100,000 individuals between 2011-2016. The majority of GEP NET are localized in the small intestine (33%), the appendix (30%), and the pancreas (12%). The most common site of GEP NEC is the pancreas (28%) (25).

### 3.4 Norway

The incidence of NEC of gastrointestinal tract in Norway increased over 2-fold between 1993-2021 and between 2017-2021, the most common NET tumors were small intestine (23%), lung (19%) appendix (13%) and pancreas (12%). The authors observed a stabilization or even a decrease in the incidence of several sites (e.g. stomach, rectum) during the last 5 years compared to the previous 5-year period (27).

### 3.5 Iceland

The total mean annual GEP NET incidence rate was 3.39 per 100,000 in 1985-1999 and 3.85 per 100,000 in 2000-2014. The most common primary tumor was found to be the appendix (32%), followed by the jejunum/ileum (24%) and the stomach (17%) (28).

### 3.6 The United Kingdom

As reported by NHS England, between 1995 and 2018, the age-adjusted NEN incidence rate increased by a factor of 3.7, from 2.35 cases per 100,000 people to 8.61 cases per 100,000 people. The incidence has increased significantly in the past 23 years. The most common sites were small intestine (1.46 per 100,000), pancreas (1.00 per 100,000) and appendix (0.95 per 100,000) (31). Another study showed a GEP NET incidence of 4.6 per 100,000 from 2013 to 2015 (26).

### 3.7 China

TIn 2017, the age-standardized incidence rate of neuroendocrine neoplasms in China was 1.14 per 100,000 individuals. The incidence rate was higher in males than in females (1.42 per 100,000 vs. 0.86 per 100,000), and higher in rural than in urban areas (1.28 per 100,000 vs. 1.05 per 100,000). The GEP NET incidence rate is 0.8 per 100,000 in 2017. The most common primary sites of GEP NET in China are the pancreas, stomach and rectum (29).

### 3.8 Japan

As indicated by data from the National Cancer Registry of Japan, the incidence of GEP NEN in Japan in 2016 was 3.53 per 100,000 individuals. Of these cases, rectal NEN constituted 53% of the total, followed by pancreas (20%) and stomach (30). There are large differences between Japanese and Western GEP NET, largely attributable to the prevalence of MEN-1 in non-functioning pancreatic endocrine tumors (38).

## 4 Genetics of GEP NEN

Two genetically distinct forms of GEP NEN have been identified: well-differentiated NET and poor differentiated NECs. NECs are characterized by TP53 and Rb1 inactivation, which is associated with a poor prognosis. In contrast, NET exhibit a wide range of molecular alterations. While GEP NET may originate from multiple sites, including the duodenum to the rectum, most molecular studies have primarily focused on two broad categories of tumors: PNET and SiNET.

### 4.1 Pancreatic NET(PNET)

#### 4.1.1 MEN1

MEN1 is the most frequently mutated and studied gene in PNET. The germline mutations in this tumor suppressor gene cause multiple endocrine neoplasia type 1 (MEN 1). A review of the literature reveals that, by the age of 50, over 90% of MEN 1 patients have one or more forms of endocrine malignancies, with the majority of cases involving parathyroid tumors (>80%), pancreatic endocrine tumors (80%-100%), anterior pituitary tumors (54%-65%), and adrenal adenomas (27%-36%) (39). Age-related prevalence of MEN1 surpasses 50% at age 20 and 95% at age 40 in all clinical features (40). The observed increase in prevalence with age may be attributed to the ontogenesis of MEN1, which is consistent with the two-hit mutation hypothesis proposed by Knudson during his study of the Rb1 gene (41, 42).This hypothesis posits that the loss of the remaining wild-type copy at the mutated MEN1 allele at the disease locus is a key mechanism underlying the disease’s pathogenesis. Since loss of heterozygosity has been identified in 86% of macrotumors, 100% of microadenomas, and, surprisingly, 95% of monohormonal endocrine cells clusters, it has been studied as a potential marker for neoplastic growth (43). Given that MEN1 is an autosomal dominant disorder, it is not anticipated that there will be a significant difference in the incidence between the genders. However, there have been reports indicating a greater prevalence of female patients (44, 45). Further validation may be required to fully comprehend this phenomenon. The MEN1 mutations play a significant role in the development of hereditary tumors in patients with MEN1 syndrome. Additionally, this mutation is the most prevalent genetic event identified in sporadic PNET, occurring in more than 35% of sporadic PNET patients in a somatic way (43). Whole exome sequencing has demonstrated that MEN1 somatic mutations are present in 40-56% of sporadic PNET, representing a considerably higher frequency than that observed for any other single gene within these tumors (46). It is noteworthy that a recent study demonstrated that allelic deletions in MEN1 are two to three times more prevalent than mutations in MEN1, aneuploid individuals exhibit more significant roles than those with single-gene mutations (46, 47). This phenomenon can be attributed to the inactivation of the MEN1 gene or the deletion of other tumor-suppressor genes located on chromosome 11, band q13.

Menin is a 68 kDa protein encoded by MEN1 gene, serving as a crucial scaffold protein. In the nucleus, menin interacts with diverse proteins to regulate gene transcription and cellular signaling pathway. For example, menin interacts to JunD and inhibits its transcriptional activity, while it binds to Smad3 to enhance TGF-β and BMP signaling pathways, thereby showing their proliferating inhibitory effects (48, 49). Menin interacts with death-domain-associated protein (DAXX) to inhibit the proliferation of NET cells, enhancing the expression of membrane metallo-endopeptidase (MME) in a synergistic manner. The menin T429K mutation, however, disrupts binding to DAXX, eliminating its MME suppression effect and promoting NET cell proliferation (50). Menin also works together with Phosphatase and Tensin homolog (PTEN), which negatively controls PI3K-Akt-mTOR pathway to suppress tumorigenesis. Studies have indicated that, in contrast to mice with single gene deletions, mice with dual knockouts of both the MEN1 and PTEN genes exhibit a more rapid development of well-differentiated G1/G2 PNET (51). This observation underscores the significance of the Menin-PTEN crosstalk. Furthermore, MEN1 has been shown to inhibit mTOR signaling, which in turn promotes lipid peroxidation and ferroptosis. This process is known to be involved in a number of different cancers (52). Given its role in the mixed-lineage leukemia histone methyltransferase complex, it is unsurprising that menin plays a pivotal role in epigenetic regulation. Furthermore, research has demonstrated that hypermethylation of numerous potential tumor suppressor genes is a common occurrence in MEN1-associated PNET, including CDCA7L and RBM47 (53). Histone modification H3K4me3 is predominantly present in the promoter region in close proximity to the transcription start site, where it serves to trigger gene transcription. In endocrine pancreas, menin directly cooperate with the promoter of p27 and p18, known as cyclin-dependent kinase inhibitors. Study showed that menin increase the methylation of H3K4me3 and promote the expression of p27 and p18 to suppress cell proliferation (54). Menin can also enhance the H3K9me3 levels at the MME promoter to suppress PNET when cooperate with DAXX (51).

#### 4.1.2 mTOR

The mammalian target of rapamycin(mTOR) is a serine threonine kinase that encoded by the mTOR gene. And mTOR locate downstream of PI3K/AKT signaling pathway, which mediate several basic cellular functions. The regulation of mTOR pathway involves upstream regulatory proteins (such as PTEN and PI3K) and downstream effectors (including MDM2, FOXO, and GSK-3β), which also regulated by diverse other signaling pathways (55). Dysregulation of PI3K/AKT signaling lead to tumorigenesis through increasing protein expression, cell migrating and promoting angiogenesis (56). For example, mutation in PTEN cause many cancers, such as breast, colon, lung, prostate (57).

**Figure 2:**
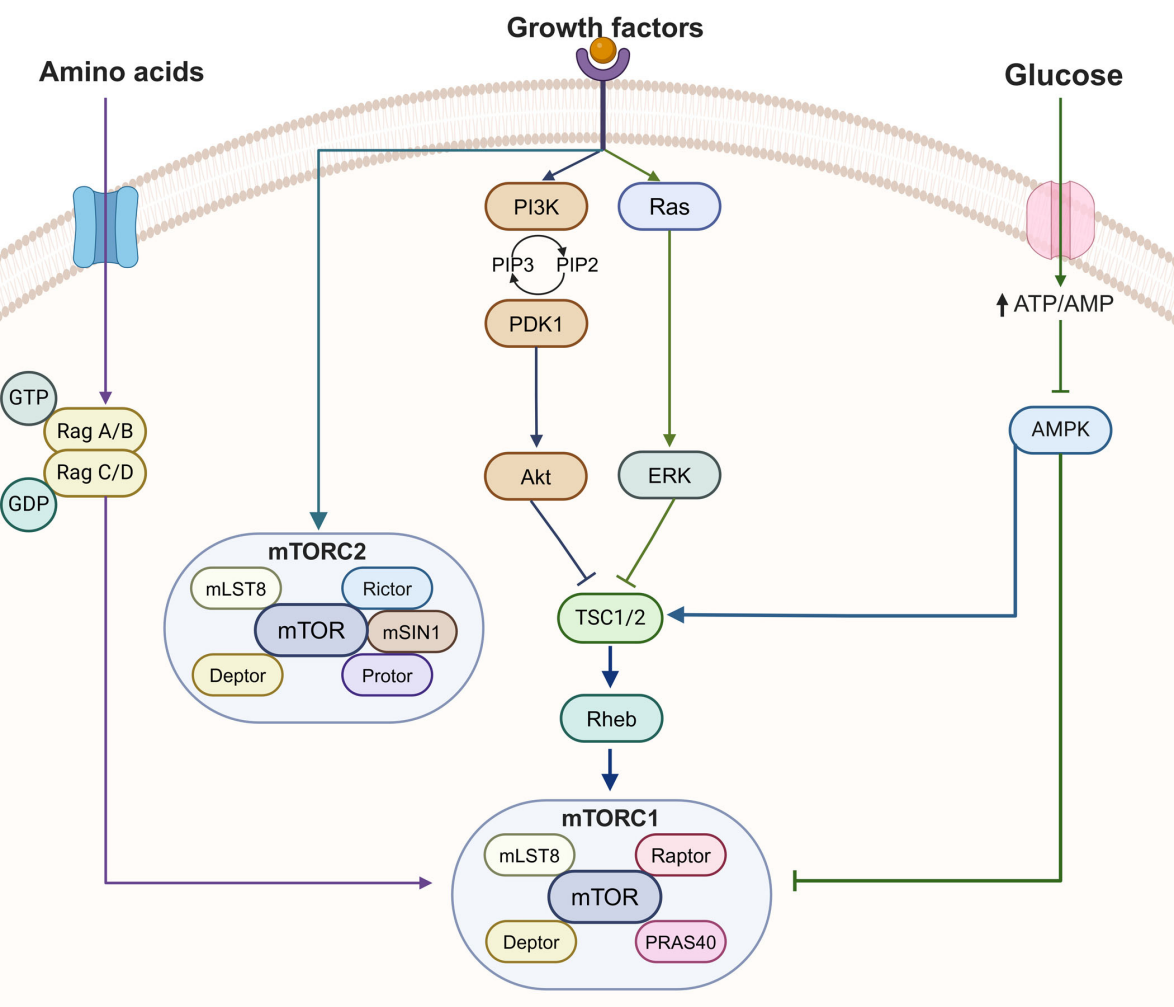
The mTOR signaling pathway. mTORC1 is linked to 3 input signals, whereas mTORC2 is controlled by a growth factor. AMPK, AMP-activated kinase; ERK, extra-cellular regulated kinase; deptor, DEP-domain-containing mTOR interacting protein; mLST8, mammalian lethal with Sec13 protein 8; mTORC, mammalian target of rapamycin complex; PDK1, phosphoinositide-dependent kinase 1; PI3K, phosphatidylinositol 3-kinase; PIP2, phosphatidylinositol bisphosphate; PIP3, phosphatidylinositol triphosphate; PRAS40, proline-rich Akt1 substrate 1; Rheb, Ras homolog enriched in brain; mSin1, stress activated protein kinase interaction protein 1; protor, protein observed with Rictor-1/; TSC1/2, tuberous sclerosis complex1/2.

In PNET, aberrant activating of PI3K-Akt-mTOR pathway involves both familial and sporadic PNET (**Figure 2**). Mutation in the TSC1 and TSC2 genes cause tuberous sclerosis (TS), characterized by benign hamartoma, cognitive impairment and epilepsy. There is also a higher risk in TSC patient for developing malignancies, including renal cell carcinoma, breast cancer and thyroid cancer (58). The development of PNET in patients with TSC is relatively common, primarily attributed to TSC2 mutations identified through genetic analysis (59, 60). The protein, tuberin, encoded by TSC2 gene, could facilitate Rheb GTP hydrolysis, leading to the inhibition of mTORC1 activation (61). It is well-established that aberrant activation of the mTOR pathway plays a significant role in sporadic PNET. The initial whole-exome sequencing (WES) study of 68 sporadic PNET cases revealed that 8.8% of cases had TSC2 mutation and 7.3% of cases had PTEN mutations (62). Additionally, a case with a PIK3CA missense mutation was also identified in this study (62). Furthermore, an inactivating mutation of DEPDC5, a tumor suppressor gene in the mTOR signaling pathway, has been recently discovered (63).

In addition to mutations in mTOR-related genes, altered expression of pathway members is common in PNET patients. RT-PCR and immunohistochemistry results revealed that TSC2 and PTEN were downregulated in sporadic PNET, which was significantly related to disease progression and shorter overall survival (64). In normal islet cells, PTEN primarily exhibited in a nuclear pattern. However, a study showed that 19 of 23 sporadic PNET correlated with abnormal high cytoplasmic expression (65). Compare to normal tissues, miR-144/451 is significantly overexpressed in insulinomas, which, in turn, promotes β-cells proliferation by up-regulating the PTEN-Akt pathway (66). Overall, these results demonstrated that a significant proportion of PNET are naturally dysregulated in the PI3K/Akt/mTOR pathway.

#### 4.1.3 VHL

Von Hippel-Lindau (VHL) syndrome, an autosomal dominant tumor syndrome, is caused by a germline mutation in VHL tumor suppressor gene. Patients with VHL are at an increased risk for developing hemangioblastoma of the central nervous system, renal angiomas, renal cell carcinoma, pheochromocytoma and pancreatic lessions (including PNET) (67). VHL encodes a 232-amino acid protein, pVHL. Under normoxic conditions, pVHL binds to hypoxia-inducible factor 1-alpha (HIF1-α), undergoing proteasomal degradation after polyubiquitination (68). During hypoxia, HIF 1-α translocates to the nucleus and interacts with HIF-1β to serve as a transcription factor, leading to increased proliferation (PDGFR and EGFR), angiogenesis (VEGF), glycolysis (CAIX and GLUT1) and Mesenchymal-epithelial transition (MET) (OCT4) (69).

**Figure 3:**
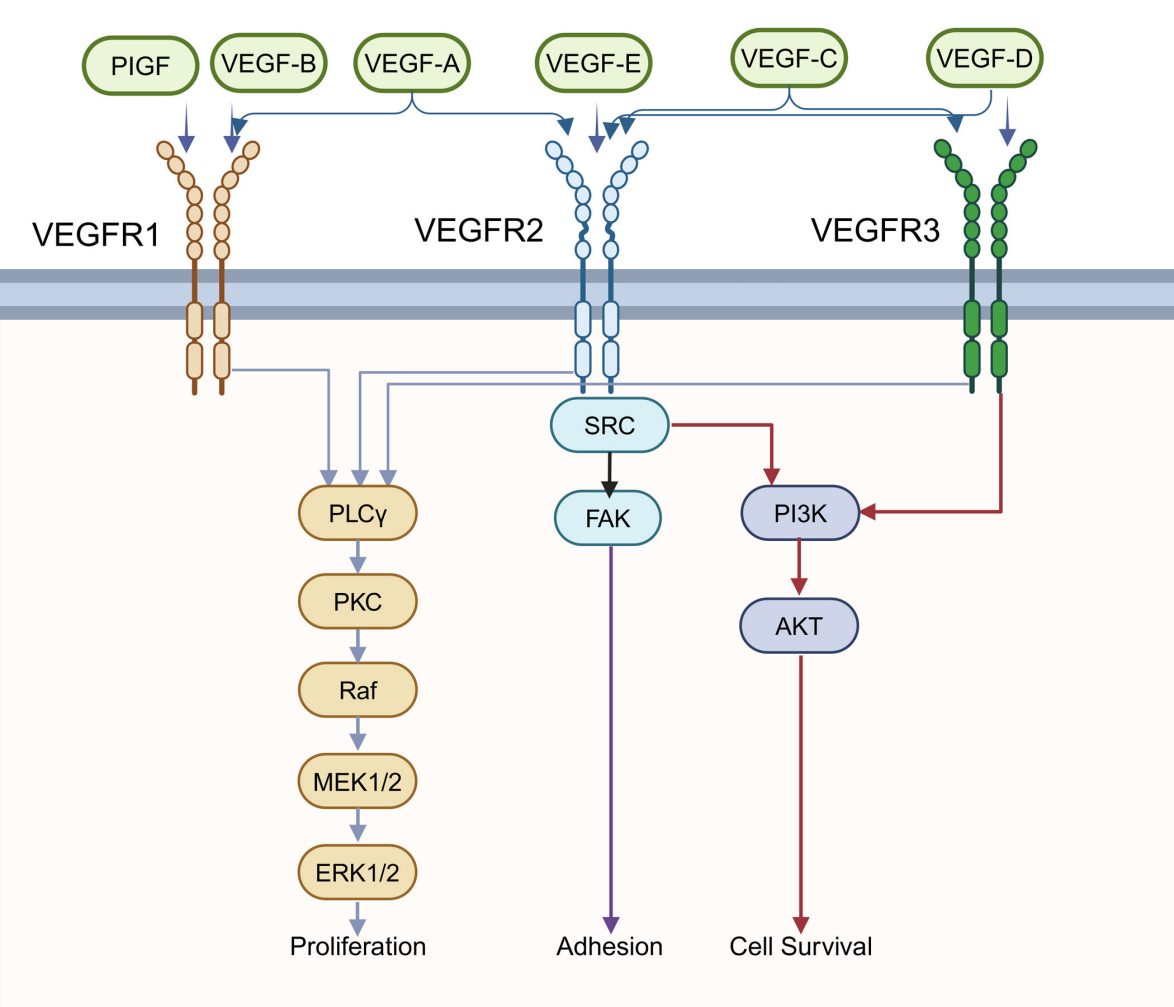
The VEGF signaling pathway. The Vascular endothelial growth factor (VEGF) family has 6 ligands (VEGF-A, B, C, D, E, placenta growth factor [PlGF]) that bind specifically to the VEGF receptor to activate different signaling pathways. The phospholipase C isoform-γ (PLCγ)–protein kinase C (PKC) pathway activates Raf protein kinase, and then the downstream Mitogen- activated protein kinase kinases 1 and 2 (MEK1/2), extra-cellular regulated kinase 1/2 (ERK1/2), will be activated sequentially, which can eventually control proliferation. Phosphoinositide 3-kinase (PI3K)/AKT signaling pathway regulates cell survival. In addition, adhesion is associated with focal adhesion kinase (FAK).

Deletion of pVHL restricts the degradation of HIF-1α in patients with VHL syndrome. Consequently, HIF target genes are upregulated, leading to tumorigenesis. Research has demonstrated elevated expression levels of VEGF in PNET (70, 71). Immunostaining results have shown that the majority of PNET express another member of the VEGF protein family, VEGF-C, at moderate to high levels. The expression of VEGF-C in uncertain or low-grade malignant PNET is relatively higher compared to benign PNET, suggesting its role in mediating tumor progression (72). Scientists have also identified VEGFR-2 as the major VEGF-C receptor highly expressed in endothelial cells of all lesions examined, highlighting its role in angiogenesis in PNET (72). In addition, VEGF family protein can also transduce autocrine signals necessary for proliferation, survival and cell migration (73, 74). These examples illustrate significant roles in regulating one of the cancer-related pathways, the VEGF pathway (**Figure 3**). Another research revealed that patients with VHL gene promoter hypermethylated also showed active hypoxia signals and related to poor prognosis (75).

#### 4.1.4 NF1 and RAS-MAPK pathway

Approximately 1/3000–4000 people worldwide are affected by neurofibromatosis type 1 (NF1), an autosomal dominant disorder. It is caused by NF1 germline mutation, which is a cancer suppressor located on chromosome 17q11.2. Patients with NF1 are predisposed to cancers of gastrointestinal tract, liver, lung, thyroid, ovary and breast. Scientists found that up to 10% NF1 individuals develop PNET, most commonly periampullary duodenal somatostainomas, pancreatic somatostinomas, gastrinomas or insulinomas (76). NF1 encodes a RAS GTPase-activating protein, neurofibromin, which function as a negative regulator of RAS/MAPK pathway and inhibitor of mTOR (77). Therefore, loss of neurofibromin results in aberrant activation of RAS/MAPK pathway, which mediate proliferation, survival, differentiation and metabolism in normal cell and often dysregulated in cancer.

Notably, regulated RAS/MAPK signaling is crucial for PNET cell survival and growth. Research revealed that 47 out of 422 cases exhibited copy number loss of HRAS (78). Additionally, KRAS was found to have a higher mutation rate in metastatic species compared to primary PNET, and is associated with metastasis and poor prognosis (78). It is interesting to find out that methylation of negative regulator of RAS/MAPK pathway, RASSF1A, is related to its low expression, thus decrease its roles in inhibiting cell growth.

#### 4.1.5 DAXX and ATRX

In the first whole-exome study of PNET, researchers reported two novel frequently mutated genes, alpha-thalassemia X-linked intellectual disability syndrome (ATRX) and death-domain-associated protein (DAXX) (62). In 68 cases, Jiao et al. identified that 17.6% of patients had ATRX mutations and 25% had DAXX mutations (62). ATRX is a heterochromatin component that belongs to the SNF2 family of chromatin remodeling proteins, regulating gene expression through chromatin modification (79). DAXX is a histone chaperone that specially interacts with H3.3. DAXX is recruited by ATRX to form heterodimers and the ATRX/DAXX complex coordinates the deposition of histone H3.3 at the pericentromeric and telomeric heterochromatin, mediating chromatin remodeling and stabilize telomere length (80, 81). In most PNET, ATRX and DAXX mutations occurs in a mutually exclusive manner, consistent with their co-function in the same pathway (62). In addition, inactivation of ATRX/DAXX complex lead to chromosomal instability and the alternative lengthening of telomeres (ALT). Telomere-specific fluorescence *in situ* hybridization (FISH) was carried out in 41 PNET by Heaphy et al. Results demonstrated that PNET with ATRX/DAXX mutation displayed aberrant telomere signals, indicating telomerase independent telomere modification, known as ALT (82). Interestingly, several studies found that ATRX/DAXX also play crucial roles in methylation regulation in genes, such as RASSF1 and PTEN (83, 84). For example, DAXX is recruited by combination of p53 and RASSF1A to involve in RASSF1A methylation and inactivation, regulating stability of murine double minute 2 (MDM2). And the methylation level is strictly controlled by DAXX expression (83).

The significance of ARTX/DAXX mutation in prognosis also has been highlighted. Chan el at. found that ARTR/DAXX mutant PNET have the characteristic of alpha cells’ gene expression, indicating a worse outcome (85). Several studies showed that ARTX/DAXX loss is positively correlated with tumor grading and staging, disease recurrence, and survival rate (85–88). However, Jiao el at. hold the opposite opinion that patients with ATRX/DAXX mutation have better overall survival than patients with wild gene type. This difference may due to selection of patient pool, and additional study include larger and varied patient are necessary (62).

### 4.2 Small intestinal neuroendocrine tumor (SiNET)

Compared to PNET, mutation analysis of SiNET is less revealing. In exosome and genome sequencing, CDKN1B was identified as the most frequent recurrent mutations in 9% of SiNET patients (89). Another gene, APC, mutation was identified in 23% carried APC mutation in 30 SiNET patients, and recent research confirmed the finding as 8% cases carried APC mutation in 52 sporadic primary SiNET. Notably, although the modest frequency of genomic disturbances, 50% of SiNET hold diver mutations in common tumor suppressor gene and proto-oncogenes (90). However, mutation only account for less than 25% cases, although they can also exert effects on haplounder-deficient genes through without the need for a “second blow”. It seems that SiNET tumorigenesis is more dependent on chromosomal alterations and aberrant methylation instead of mutations.

The whole exome sequencing data demonstrated the duplicate loss of chromosomes 11 and 18, as well as the increase in chromosomes 4, 5, 14, and 20 (91, 92). The fact that loss of chromosome 18 is the most frequently events in SiNET led to further research into Smad2 and Smad4, which are tumor suppressor gene located on chromosome 18. However, scientists didn’t find relevant expression loss of Smad2 and Smad4 (92). Therefore, additional researches are necessary to explore the function of chromosome 18 loss in SiNET.

Enrichment analysis revealed overactivation of MAPK, mTOR and Wnt pathways. Notably, members in PI3K/mTOR play crucial roles, induced by amplification pf EGFR, HER2 or PDGFR (93). Methylation of RASSF 1A was identified in 32% SiNET cases and it can promote proliferation through cell cycle control and semaphorin 3 inactivation. Meanwhile, there are unexpected effects of SEMA 3F methylation and, thus, its product, semaphorin 3 loss. Researchers reported that semaphorin 3 loss can disinhibit PI3K and mTORC, representing a possible resistance mechanism to everolimus (94). Furthermore, the expression of semaphorin 3 is correlated with higher tumor stage (94). MiRNA upregulation and downregulation were identified in metastatic SiNET patients. The most consistent findings included the upregulation of miR-96, -182, -183, -196a and the downregulation of miR-1, -31, -129-5p, -133a, -215, miR-143-3p, and miR-375 (95–97). These differences in microRNA expression are clinically utilized as predictors of overall patient survival and miR-375 was identified as the strongest one (98).

## 5 Treatment

### 5.1 Surgery

Variation in the surgery of GEP NEN depends on the site and type of tumor. Major conferences and guidelines recommend surgery for most GEP NEN patients (99).

However, pancreatic neuroendocrine carcinoma (PNEC) has a poor prognosis and are not amenable to surgical resection (100). But even in those patients for whom there is no possibility of cure, surgery may be necessary in case of acute life-threatening complications (101).

Surgery is the first line of treatment for PNET and the only treatment for locally advanced NET, 70-90% of cases can be cured with surgery (102, 103).

PNET are clinically classified as either non-functional or functional, and functional PNET are supposed to be evaluated for surgery (104, 105). The NANETS guidelines recommend pancreatectomy for tumors larger than 2 cm for non-functional PNET, if there is localized functional PNET without distant metastases, resection is recommended (106).

The types of pancreatic surgery vary from “typical” to “atypical” resections, depending on the tumor burden, but laparoscopic resection is used most often (107–109). Patients in good general condition can undergo pancreas-sparing pancreatectomies, which can decrease the incidence of pancreatic insufficiency (104). The use of endoscopic ultrasound-guided therapies can be considered as an alternative treatment for patients for whom surgery is not available at the low-grade level of less than 20 mms PNEN (110–112). Minimally invasive pancreatectomy is technically feasible and safe and has advantages in terms of postoperative recovery (109, 113, 114). Furthermore, robotic distal pancreatectomy is also safe and effective (108).

Gastric NET can be divided into three subtypes based on clinical and histological features (115, 116). Treatments need to be selected based on the type of gastric NET. The treatment of type 2 gastric NET is generally similar to that of type 1. Survival of type 1 after endoscopic surveillance or surgical resection is high (117). Type 3 is more malignant and more likely to metastasise than other gastric NET types (118, 119). The choice of endoscopic mucosal resection, endoscopic submucosal dissection or surgery is based on the number of lesions and whether or not the invasion of the muscularis propria occurs (20, 120). The Nordic guidelines and the ENETS guidelines recommend surgical resection with lymphadenectomy similar to that performed for gastric adenocarcinoma (20, 120, 121). Chinese guidelines and a review point to endoscopic resection as an alternative for patients for who are not candidates for surgery, but there is a higher risk of lymph node spread (118, 122).

SiNET are frequently found in the ileum, small intestine resection with removal of lymph nodes is recommendable for SiNET (123, 124). ESMO guidelines recommend surgery for locally advanced SiNET because the presence of a large mesenteric mass can lead to intestinal obstruction and/or ischaemia (evidence level V, recommendation level B) (125). Duodenal neuroendocrine tumors(dNEN) have a high incidence of lymph node metastasis but have a positive surgery prognosis (126). In patients with colorectal NET who have predictive factors for lymph node metastasis, surgical resection with lymph node dissection is an option (127). Conventional imaging has been demonstrated to have a low detection rate of localized regional lymph nodes and micrometastases in preoperative dNEN, with understaged cases present in 38%. It is recommended that endoscopic ultrasound be included as a preoperative tool to obtain more accurate local staging (128).

In select cases of smaller colonic NET G1 (typically less than 10 mm), endoscopic resection may be a viable option, as recommended by the 2023 ENETS guidelines (129). In the treatment of rectal neuroendocrine tumors, endoscopic or surgical technique should be selected based on the size of the tumor. Endoscopic resection is the recommended course of action for lesions measuring 10 mm or less in diameter, as it has a relatively low recurrence rate (130–132). For lesions between 10-20 mm, a comprehensive imaging assessment should be conducted and deliberated by a multidisciplinary team (MDT) to ascertain the optimal course of action, which may entail endoscopic resection or surgical intervention (129). For lesions exceeding 20 mm, surgical resection, including low anterior resection or abdominal perineal resection, is advised following the exclusion of unresectable distant metastases (133).

Most appendiceal neuroendocrine neoplasms (aNEN) can be treated with simple appendectomy or right hemicolectomy. Patients with aNEN >2 cm are recommended for right hemicolectomy, while patients with aNEN <1cm could undergo appendectomy (134). The treatment of aNEN with diameters of 1-2 cm is controversial, but a recent study noted that right hemicolectomy is unadvisable after the complete removal of a 1-2 cm aNEN by appendectomy (135).A lot of people with NET develop liver metastases, cytoreduction can be an option when NET liver metastases are resectable in at least 70% of patients (136).

In advanced stages of NEN, surgical intervention has been shown to confer benefits to a subset of patients (101). The findings of the study indicate that surgical resection can be advantageous for patients with NEN G3 and mixed neuroendocrine-non-neuroendocrine neoplasms (MiNEN) (137).

However, a single-center study of 615 patients with SiNET revealed a significant risk of recurrence following intended radical surgery (138). In another study, 441 patients were included, of whom 224 had PNET and 217 had SiNET. The results demonstrated that approximately 30% of patients with enteropancreatic NET experienced recurrence within five years of radical surgery (139).

Surgical approaches to GEP NEN vary widely according to primary tumor site, tumor classification and lesion size, and each NEN requires dedicated assessment to determine the relevant characteristics of GEP NEN (140).

### 5.2 Somatostatin analogs (SSAs)

SSAs have high affinity for STTR2 and moderate affinity to SSTR5, which can be used to control the symptoms of hormone overproduction, especially in functional metastatic PNET (141–144). Five subtypes of SSTR receptors have been identified. SSTR receptors belong to the family of G protein-coupled receptors, and more than 70% of NET tumor cells overexpress SSTR type 2 and 5 (145–147). SSTR5 is expressed in somatostatinomas and SSTR2 is expressed in gastrinomas and glucagonomas in the functional PNET (147).

Positive somatostatin receptor imaging is required when applying SSAs for antiproliferative effects (148, 149). SSAs also have antiproliferative effects, cell cycle inhibition and an increase in apoptosis (150). The results of the PROMID, CLARINET and CLARINET FORTE clinical studies showed that for in midgut NET, SSAs could lengthen time to tumor progression, and in progressive GEP NETs could improve progression-free survival (143, 147, 151–154). Two SSAs are currently available that have been approved for GEP NET treatment in the United States, octreotide and lanreotide, with both being long-acting (144, 155). Both SSAs were well tolerated and had a very low-adverse reaction rate (149). No evidence exists to suggest that the two SSAs differ in controlling hormone secretion and tumor growth (142). The initial prospective study to assess the efficacy of growth inhibitor analogs in MEN1-related PNET revealed that lanreotide demonstrated efficacy as an antiproliferative therapy for MEN1-related PNET with a diameter of less than 2 cm (128).ESMO,NANETS,ASCO guidelines recommend SSAs as first-line treatment for functional NET or STTR-positive or metastatic high-differentiated GEP NET (125, 156, 157). However, patients can become resistant to SSAs treatment, and the exact mechanism is unclear (158).

Second generation SSAs is now available for the treatment of other diseases with a greater affinity for SSTR5, and may be used in the future for the treatment of GEP NEN (159, 160).

The most commonly reported adverse effects associated with SSAs are gastrointestinal events (diarrhea and constipation), abdominal discomfort, and the formation of gallstones (161).

### 5.3 Peptide receptor radionuclide therapy (PRRT)

The somatostatin receptors (SSTR) is commonly expressed in GEP NEN, especially in well-differentiated NET, whereas it is not expressed in normal tissues, and radioactive peptides can be used to label this receptor, which allows the radionuclides to enter the tumor tissue and eventually kill the tumor cells (17, 162–164). The use of highly active SSTR-binding ligands is now commonly referred to as peptide receptor radionuclide therapy (PRRT) (165). SSTRs receptor number is directly related to the therapeutic efficacy of PRRT (166). Somatostatin receptors are rarely expressed in NEC, making PRRT less suitable for NEC.PRRT is valid and secure in the treatment of NET (167–172). The results of the NETTER-1 trial showed that 177Lu-DOTATATE was more effective, and progression free survival was significantly improved compared to the use of high-dose octreotide (173). It is noteworthy that the NETTER-1trial exclusively included patients with SiNET and excluded those with PNET.

PRRT may also be effective in patients with G3 NET, but this needs to be validated with more evidence from clinical studies (174). A large retrospective study showed that PRRT treatment was effective in the treatment of NET G3, and in this study PRRT treatment showed promising response rate and disease control rate (175). The results of the NETTER-2 trial indicated that PRRT is an appropriate treatment option for patients with advanced Grade 2-3 GEP NET. The combination of ^177^Lu-Dotatate and octreotide 30 mg long-acting repeatable (LAR) demonstrated a 72% reduction in the risk of disease progression or mortality compared to high-dose octreotide 60 mg LAR (176).

Not all patients will benefit from PRRT, and it is necessary to investigate ways to improve the effectiveness of PRRT. Using alpha-emitters, PRRT in combination with chemotherapy, these methods may have more effective outcomes (177–180). However, attention should also be paid to the potential for greater toxicity associated with combination therapy (179) (**Table 4**).

**Table 4:** Clinical trials investigating PRRT in combination with other treatment.

Clinicaltrials.gov NCT Number	Interventions	Cancer type	Phase	Study Start
NCT05610826	Surgery + PRRT (Cytoreduction+Lu-177 dotatate)	PNET	1/2	7-Mar-23
NCT05249114	TKI + PRRT (Cabozantinib+Lu-177 dotatate)	Progressive, Previously Treated, SSTR2 Positive NET	1	28-Dec-22
NCT05870423	PARP inhibitor+ PRRT (Olaparib+Lu-177 dotatate)	Well-differentiated advanced GEP NET	1	1-Jun-22
NCT05053854	PARP inhibitor+PRRT (Talazoparib+Lu-177 dotatate)	Metastatic pancreatic or midgut NET	1	8-Dec-21

GEP, Gastroenteropancreatic; NEC, neuroendocrine carcinoma; NET, neuroendocrine tumor; PRRT, peptide receptor radionuclide therapy; SSTR, somatostatin receptors; PARP, poly ADP-ribose polymerase.

The first generation of radionuclide gamma-emitting 111Indium had a weak cytotoxic effect (181). Second-generation radionuclides include beta-emitting 90Yttrium (90Y) and 177Lutetium (177Lu), which have better therapeutic effects killing nearby tumor cells (170). The alpha-emitters have been the focus of current research due to their higher energy and greater tendency to cause DNA breaks. The alpha-emitters used in NEN patients are 213Bi, 225Ac, 212Pb (182). Current phase I clinical trials have shown [212Pb] Pb-DOTAMTATE to be well tolerated with fewer adverse effects (183).

The NANETS guidelines and ASCO guidelines recommend PRRT as a second-line treatment for metastatic intestinal NET G1/G2 if positive for SSTR expression (157, 180, 184). ESMO guidelines recommend PRRT as second-line treatment for midgut NET patients with progressive disease after SSA therapy [I, A] (125, 156). The delivery mode of PRRT also can change the therapeutic effect. PRRT arterial delivery to the hepatic artery may lead to improved results in the treatment of NEN with hepatic metastases (185). PRRT retreatment after initial PRRT did not negatively affect safety (186). The results of the meta-analysis demonstrated that Salvage PRRT exhibited a lower objective response rate (17.6% vs. 38.6%, P < 0.001) and a lower disease control rate (77.6% vs. 99.7%, P < 0.001) compared to the initial treatment (187).

A cohort study indicates that the use of upfront PRRT in patients with enteropancreatic NET who have experienced disease progression with SSA treatment was associated with improved progression-free survival outcomes compared with upfront chemotherapy or targeted therapy (in unmatched populations 2.5 years vs. 0.7 years; *P* < .001]) *(* 188). Furthermore, a review of the literature indicates that patients with PNEN may benefit from PRRT as a neoadjuvant approach (189).

The side effects of PRRT are nausea, vomiting, myelosuppression and abdominal discomfort (125, 190–192). The incidence of Grade 3/4 toxicity in any of the blood counts was less than 15% in the treated patient (192). A retrospective study of 807 patients revealed that renal toxicity was observed in 35% of cases, with Grade 3/4 toxicity occurring in 1.5% (193).

Strategies may be employed to customize PRRT for each patient, including considerations such as patient selection, dosimetry, combination therapies, and modification of the therapeutic index (194).

### 5.4 Targeted therapy

Currently, VEGF and mTOR targeting drugs have been applied in the treatment of NET, while other targeting drugs such as Hypoxia-inducible factor (HIF) inhibitor, Cyclin dependent-kinase 4/6 inhibitors are under investigation (125). The use of targeted drugs in combination with immunotherapy in NET is also a hot topic of current research (195).

#### 5.4.1 Mammalian target of rapamycin (mTOR) pathway

The mTOR inhibitor everolimus has been used in the treatment of NET. Guidelines recommend that everolimus can be used in SSTR-negative G1-G2 and advanced PNET (20, 157). The RADIANT-4 study demonstrated that treatment with everolimus improved the progression-free survival of GEP NET and was better tolerated, while also exhibiting strong antitumor activity (196). The combination of everolimus and SSA may be considered for routine treatment of patients with functional NET in the future (197). A new mTOR inhibitor, nab-Sirolimus, is under investigation. It has been found discovered that nab-Sirolimus has superior target inhibition when compared to oral mTOR inhibitors (198). A phase 2 clinical trial is now investigating the effect of nab-Sirolimus in the well-differentiated, advanced inoperable metastatic GEP NET(NCT05997056). Other mTOR inhibitors, such as sapanisertib, could play an important role in treating NEN and further studies are needed to assess the effect in PNET (199).

The most frequently observed adverse effects associated with everolimus therapy include stomatitis, fatigue, rash, diarrhea, hyperglycemia, and anemia (200–204). The most common Grade 3 or 4 drug-related adverse events were stomatitis (9%), diarrhea (7%), infections (7%), anemia (4%), and fatigue (4%) (196).

#### 5.4.2 Vascular endothelial growth factor (VEGF) pathway

VEGF is one of the most widely studied biomarkers with the ability to induce abnormal angiogenesis in tumors, the ability of tumors to escape the immune system, invade and metastasise may be enhanced (205–207). VEGF binds to VEGF receptors (VEGFRs),VEGFR-1, VEGFR-2 and VEGFR-3 (208) (**Figure 3**).

Surufatinib can simultaneously target VEGFR-1, 2, 3 and fibroblast growth factor receptor 1, targeting multiple pathways simultaneously reduces tumor angiogenesis more effectively than targeting one pathway alone (209–211). Phase 3 clinical trials of surufatinib have demonstrated favorable therapeutic outcomes in both advanced pancreatic and extra-pancreatic NEN (212). Surufatinib has a controlled safety profile when combined with toripalimab immunotherapy and appears to have superior anti-tumor activity against NEC (213).

The most common adverse effects associated with surufatinib therapy encompass proteinuria, diarrhea, increased thyroid stimulating hormone in the blood and increased bilirubin in the blood, the most prevalent grade 3 or worse adverse events were hypertension (36%) (212).

Sunitinib is a multitarget tyrosine kinase inhibitor that targets VEGF and platelet-derived growth factor (214). Sunitinib demonstrates efficacy in patients with PNET and exhibits a favorable long-term safety profile (214, 215). Other drugs that target VEGFR and have potential include Cabozantinib, Lenvatinib, Nintedanib, Anlotinib (216, 217).

The most prevalent adverse effects associated with sunitinib therapy include fatigue, gastrointestinal intolerance, and dermatitis. Additionally, serious toxicities, classified as Grades 3 and 4, have been observed in 4 out of 41 cases (218).

In a hypoxic environment, HIF-1α induces VEGF expression, which may cause angiogenesis in tumors (219). Belzutifan is the first HIF inhibitor and the first FDA-approved systemic therapy for VHL-related tumors (219, 220). In VHL disease, the incidence of PNET ranges from 9% to 17% (221).The 2021 edition of the NCCN guidelines recommends the use of belzutifan for the treatment of G1 and G2 locoregional advanced metastatic progressive PNET if the VHL gene is mutated (222). Future drug combination strategies may lead to a better prognosis for patients, with simultaneous inhibition of MTOR and VEGF pathways showing promise in the treatment of NEN (216, 223).

The most commonly reported side effects of Belzutifan include anemia and hypoxia-related symptoms (224, 225). In a phase 1 trial include 43 patient A total of 31 patients (72%) experienced treatment-related adverse events of any grade, while 8 patients (19%) across all dose levels experienced treatment-related adverse events of grade 3 and 4 (226).

#### 5.4.3 Cyclin dependent-kinase 4/6 inhibitors

Cyclin dependent-kinase 4/6 inhibitors, has promising potential for treatment in NEN and trials are already underway. However, a multicenter, phase II study reported that the combination of Ribociclib and Everolimus was not effective and may be toxic in well-differentiated foregut NET. One trial of the role of Abemaciclib’s anti-tumor activity in metastasized inoperable GEP NET is ongoing (NCT03891784).

Cyclin-dependent kinase 4/6 inhibitors have been identified as a promising avenue for treatment in NEN, with clinical trials already underway (227). However, a multicenter, phase II study reported that the combination of ribociclib and everolimus was not effective and may be toxic in well-differentiated foregut NET (228). One trial investigating the role of abemaciclib’s anti-tumor activity in metastasized inoperable gastrointestinal GEP NET is currently ongoing (NCT03891784).

### 5.5 Chemotherapy

Chemotherapy is primarily used to treat patients with G3 GEP NET and NEC. In contrast to G1,G2 GEP NET, chemotherapy is more important in the treatment of G3 GEP NET, as a high proliferative index indicates more effective chemotherapy (229). Platinum and etoposide chemotherapy is now the first-line choice for extrapulmonary NEC (156, 230). NANETS guidelines recommend the use of fluoropyrimidine in combination with platinum as second-line treatment for extrapulmonary NEC (156). ASCO guidelines recommend CAPTEM(Capecitabine and Temozolomide) chemotherapy in patients with SSTR-negative G1-G2 PNET (157). The effects of chemotherapy in combination with other treatments are being studied in clinical trials (**Table 5**). Moreover, the study by Tafuto et al. demonstrated that metronomic temozolomide (mTMZ) as monotherapy represents a viable treatment option for patients with advanced G2-G3 NET, particularly in those with an ECOG Performance Status (PS) score of 2, exhibiting good tolerability and clinical improvement (231).

**Table 5:** Clinical trials investigating chemotherapy.

Clinicaltrials.gov NCT Number	Interventions	Cancer type	Phase	Study Start
NCT06132113	Bispecific antibody (DLL3/CD3)+ chemotherapy (BI 764532 +Carboplatin/ Etoposide/ Cisplatin)	NEC	1	20-Dec-23
NCT05879055	Bispecific antibody (PD-(L)1 and VEGF) + chemotherapy (PM8002+ FOLFIRI)	NEC and Ki-67≥55% G3 NET	2	17-May-23

GEP, Gastroenteropancreatic; NEC, neuroendocrine carcinoma; NET, neuroendocrine tumor; PD-1, Programmed Cell death 1; PD-L1, Programmed Cell Death Ligand 1; FOLFOXIRI, Fluorouracil, Leucovorin, Oxaliplatin, Irinotecan.

The side effects of temozolomide include anemia, platelet count decreased, neutrophil count decreased, fatigue, nausea, and constipation and vomiting, with Grade 3-4 toxicity rate of 22% when used alone (232).

### 5.6 Immunotherapy

Immune checkpoint inhibitors (ICIs) can be broadly classified into two groups, antibodies to programmed death 1 (PD-1) and its ligand (PD-L1) and antibodies targeting cytotoxic t-lymphocyte antigen 4 (CTLA-4), which can be monitored in many cancer types (233, 234). These have negative regulatory effects on t-cell immune function, and inhibition of these targets increases immune system activity (235). Immunotherapy has been developing rapidly recently and has promising applications in many tumors, however the efficacy of treatment in NEN is not well defined and more prospective studies are required to evaluate the value of ICI in GEP NEN therapy (236–239). However, ICIs may be a promising treatment option for high-grade, poorly differentiated NEN (240, 241).

One study indicates less PD-1/PD-L1 in the small intestine or pancreas, which may lead to poor use of ICI (242).However, one study noted that PD-1/PD-L1 expression is common in poorly differentiated NEC of the digestive system (243). One article shows limited potential for anti-PD-1/PD-L1 monotherapy for digestive NECs (244). A recent animal trial showed that the combination of PRRT and anti-PD1 provided the strongest response to NET, with an overall effect superior to that of ICI or PRRT alone (245, 246).

Pembrolizumab monotherapy has a proven safety profile in patients with advanced NEN, but has limited anti-tumor activity and further studies may be needed to identify effective combination safety profile in the treatment of high-grade NEN following first-line chemotherapy (247). The DART trial showed efficacy of ipilimumab in combination with nivolumab in non-pancreatic neuroendocrine tumors, particularly high-grade NEC (248). The primary adverse effects associated with pembrolizumab treatment include fatigue, arthralgia, edema, nausea, vomiting, and diarrhea (249, 250). Of the total 29 of cases, only 9 were classified as grade 3 events, and no grade 4 events were identified as potentially drug-related (249).

The NCCN guidelines classify the use of Pembrolizumab for the treatment of advanced or high TMB tumors as category 2B(lower level of evidence and NCCN considers the treatment to be appropriate) (222, 250). Treatment of patients with locally advanced or metastatic NET with the combination of lipilimumab and nivolumab is also recommended as an alternative to clinical trials (category 2B) (222, 251).

Investigating the microenvironment and immune mechanisms of NEN tumors can drive advances toward ICI and other combination therapies, which are critical for NEN immunotherapy (252) (**Table 6**).

**Table 6:** Clinical trials investigating ICI in combination with other treatment.

Clinicaltrials.gov NCT Number	Interventions	Cancer type	Phase	Study Start
NCT06070740	XELOX Chemotherapy + PD-(L)1 inhibitor (Capecitabine + Oxaliplatin + Durvalumab)	Gastrointestinal NEC	2	1-Nov-23
NCT05746208	TKI+ PD-(L)1 inhibitor (Lenvatinib +Pembrolizumab)	NEN G3	2	17-Jul-23
NCT05627427	TKI+ PD-(L)1 inhibitor (Surufatinib + Sintilimab)	Metastatic and Pancreatic NEN G3	2	1-Jul-22
NCT05289856	TKI+ PD-(L)1 inhibitor (Cabozantinib +Avelumab)	NEN G3	2	28-Mar-22
NCT05015621	TKI+ PD-(L)1 inhibitor (Surufatinib +Toripalimab)	Advanced NEC	3	18-Sep-21
NCT04079712	TKI+ PD-(L)1 inhibitor (Combination of Cabozantinib, Nivolumab, and Ipilimumab)	Metastatic NEN and NEC	2	6-Aug-20
NCT03980925	Chemotherapy + PD-(L)1 inhibitor (Platinum-doublet Chemotherapy+Nivolumab)	GEP NET	2	11-Oct-19
NCT03591731	CTLA-4 inhibitor + PD-(L)1 inhibitor (Nivolumab +/- Ipilimumab)	NEC	2	2-Jan-19

GEP, Gastroenteropancreatic; NEC, neuroendocrine carcinoma; NET, neuroendocrine tumor; G3, Grade 3; TKI, Tyrosine kinase inhibitors; PD-1, programmed Cell death 1; PD-L1, Programmed Cell Death Ligand 1; CTLA-4, cytotoxic t-lymphocyte antigen 4; XELOX, Capecitabine + Oxaliplatin.

### 5.7 Interferon-alpha (IFN-α)

Interferon-alpha (IFN-α) has been demonstrated to possess antiproliferative and pro-apoptotic properties in NET. IFN-α represents a second-line treatment option, employed primarily in the management of progressive or functional PNET (253, 254). In accordance with the ESMO guidelines, if a NET patient has a SSTR-negative status, IFN-α may be considered as an alternative to other treatments [IV, B] (125, 184). Treatment with IFN-α is a treatment for refractory carcinoid syndrome, but it is not well-tolerated (125, 255). The use of interferon in clinical practice has been constrained by the occurrence of adverse effects. The main adverse effects of IFN-α are fever(76%), anorexia(71%), arthralgia(52%),injection site pain(28%) and headache(14%) (256). Among these, the suppression of bone marrow function represents a significant concern associated with IFN-α (257). The incidence of side effects may be reduced with the use of weekly pegylated formulations (258). In a phase III trial, no significant difference in progression-free survival between the bevacizumab and IFN groups, indicating that these drugs exhibit comparable antitumor efficacy in patients with advanced NET (259). The combination of IFN-α with octreotide or 131I-MIBG was found to be ineffective and did not demonstrate any synergistic effects (260, 261).

### 5.8 External beam radiotherapy (EBRT)

Radiotherapy techniques have developed at a relatively rapid pace, from total abdominal irradiation to stereotactic approaches, with major improvements in side effects (262). EBRT is rarely employed for the treatment of GEP NET, and there is a paucity of published literature on this subject. Several studies have demonstrated that EBRT can be utilized as a treatment for inoperable PanNEN, resulting in favorable local control and is well tolerated (263–265). Stereotactic body radiotherapy enables the precise delivery of high doses of radiation to small targets (266).

## 6 Summary and prospects

NEN is a heterogeneous group of tumors, and GEP NEN is the most prevalent. The incidence and location of GEP NEN vary across different countries. As research into NEN has progressed, a number of genetic and pathogenic mechanisms associated with GEP NEN have been identified. These include the MEN1, mTOR, VHL, NF1, DAXX and ATRX pathways. A variety of treatment options are currently available for neuroendocrine neoplasms (NEN). Surgical intervention represents the sole treatment option for localized GEP NET, and it also plays a role in the management of metastatic NET and NEC. PRRT employs the targeted delivery of radionuclides through the utilization of the high expression of SSTR observed in GEP NEN. The current focus of research is on immunotherapy and targeted therapies. SSA has been demonstrated to be an effective agent for the control of hormone overproduction, while chemotherapy has been shown to be a valuable adjunctive treatment for G3 GEP NET and NEC. It is imperative that future research on the genetics of GEP NEN be intensified in order to identify new therapeutic targets and potentially alter treatment strategies. Combination therapy represents a promising avenue of research, but it is of the utmost importance that researchers take note of the incidence of adverse effects and drug toxicity. Accurate assessment of the patient’s condition and selection of the appropriate treatment modality can lead to personalized treatment that is more beneficial to the patient.
